# Spatial analysis and evaluation of medical resource allocation in China based on geographic big data

**DOI:** 10.1186/s12913-021-07119-3

**Published:** 2021-10-12

**Authors:** Sida Wan, Yanming Chen, Yijia Xiao, Qiqi Zhao, Manchun Li, Shuqi Wu

**Affiliations:** 1 Jiangsu Provincial Key Laboratory of Geographic Information Science and Technology, Key Laboratory for Land Satellite Remote Sensing Applications of Ministry of Natural Resources, Collaborative Innovation Center for the South Sea Studies, School of Geography and Ocean Science, Nanjing University, Nanjing, Jiangsu 210023 People’s Republic of China; 2 School of Earth Sciences and Engineering, Hohai University, Nanjing, 211100 People’s Republic of China; 3 School of Geosciences, China university of petroleum, No.66 West Changjiang Road, Qingdao, Shandong Province 266580 People’s Republic of China

**Keywords:** Medical resource scoring model, Resource evaluation, Geographic big data, Resource evaluation, Spatial analysis

## Abstract

**Background:**

Spatial allocation of medical resources is closely related to people’s health. Thus, it is important to evaluate the abundance of medical resources regionally and explore the spatial heterogeneity of medical resource allocation.

**Methods:**

Using medical geographic big data, this study analyzed 369 Chinese cities and constructed a medical resource evaluation model based on the grading of medical institutions using the Delphi method. It evaluated China’s medical resources at three levels (economic sectors, economic zones, and provinces) and discussed their spatial clustering patterns. Geographically weighted regression was used to explore the correlations between the evaluation results and population and gross domestic product (GDP).

**Results:**

The spatial heterogeneity of medical resource allocation in China was significant, and the following general regularities were observed: 1) The abundance and balance of medical resources were typically better in the east than in the west, and in coastal areas compared to inland ones. 2) The average primacy ratio of medical resources in Chinese cities by province was 2.30. The spatial distribution of medical resources in the provinces was unbalanced, showing high concentrations in the primate cities. 3) The allocation of medical resources at the provincial level in China was summarized as following a single-growth pole pattern supplemented by bipolar circular allocation and balanced allocation patterns. The agglomeration patterns of medical resources in typical cities were categorized into single-center and balanced development patterns. GDP was highly correlated to the medical evaluation results, while demographic factors showed, low correlations. Large cities and their surrounding areas exhibited obvious response characteristics.

**Conclusions:**

These findings provide policy-relevant guidance for improving the spatial imbalance of medical resources, strengthening regional public health systems, and promoting government coordination efforts for medical resource allocation at different levels to improve the overall functioning of the medical and health service system and bolster its balanced and synergistic development.

**Supplementary Information:**

The online version contains supplementary material available at 10.1186/s12913-021-07119-3.

## Background

Before the onset of the COVID-19 global pandemic, the Nuclear Threat Initiative, Johns Hopkins Center for Health Security, and the Economist Intelligence Unit released a report on health security [1]. Termed as the Global Health Security Index, this report is the first-ever comprehensive ranking of 195 countries based on responses to 140 questions and 34 indicators in six categories. The indicators show that there is scope for a fundamental improvement in health security in countries around the world. China ranked 30th worldwide with a score of 45.7/100 in the category “adequate and robust health systems to treat patients and protect health workers”, which indicates that China exhibits a good level of medical resource adequacy compared to other countries in the world. During the pandemic, China has demonstrated a remarkable ability to deploy healthcare resources, reflecting the progress and growth of the country’s healthcare sector. However, China’s rapidly growing economic model has, to some extent, contributed to public health disparities [2]. Today, China faces serious challenges in healthcare equity.

The establishment of a perfect healthcare system must be based on the equity of medical resource allocation [3]. Worldwide, government agencies and researchers have made long-standing efforts to achieve healthcare equity. In its explorations of comprehensive healthcare coverage, the World Health Organization states that all countries must seek to improve equity in the use of health services, quality of services, and financial protection for their citizens [4]. Goddard and Smith illustrated the theoretical framework of healthcare resource imbalance using real-life experiences in the UK [5], and Emanuel et al. explored the ethical value and policy recommendations for equitable distribution of medical resources during the COVID-19 pandemic [6]. Evaluations of medical resources and the quality of health services in the United States have been conducted using spatial regression methods [7]. Goddard et al. used the Gini coefficient to evaluate the balance of supply and demand and the spatial distribution of general practitioners in England and Scotland [8]. Law and Perlman focused on the mental health industry [9]. They used a shared component spatial modeling approach to analyze healthcare services and applications. Messina et al. conducted model explorations at finer scales, namely medically underserved areas, using an accessibility model, and analyzed optimal hospital locations based on a demand model [10].

Previous studies on the evaluation of medical resources pertaining to China can be roughly divided into two categories, as explained below.

1) Studies on the spatial distribution of medical resources: Most of these works considered provinces as the basic research unit while conducting the evaluation at the national level [11], while the majority of the research objects at the non-national level focused on the cities in a province [12] or a region [13]. As these situations tend to be highly influenced by regional regulation policies, these studies tended to suffer from limitations posed by circumstances at the regional level. Most of the analyzed data involved indicators, such as the number of medical institutions and number of beds in medical institutions [14] and health manpower [15]. The data were sourced from Chinese statistical yearbooks and regional statistical yearbooks to measure the abundance of healthcare resources. The majority of these works used the Gini coefficient, Thiel index, Atkinson index [15], factor analysis method, and/or entropy-weight-TOPSIS comprehensive evaluation approach [16] to evaluate the healthcare resource supply in each province and region. Policy recommendations for the equalization of medical and health services were proposed according to the results of spatial distribution analyses and influencing factors.

2) Research on the accessibility of healthcare services: These studies tended to focus on a single city or province, and unlike the cases in the previous category, the data used here were typically spatial location/point-of-interest data of medical institutions. Urban road network data were also applied to analyze the accessibility of each healthcare institution within a city [17]. These studies mainly allowed analyses of the spatial distribution of medical institutions within a city as well as their planning and construction [18]. Recently, Yin et al. explored the uneven distribution of the spatial accessibility of healthcare facilities nationwide [19]. These results enriched the literature to some extent.

In the aforementioned studies, the data analyzed were mostly sourced from the medical statistics yearbooks of Chinese provinces and cities. The data sources were relatively simple, and the foci of the research were primarily limited to certain levels (provincial and municipal). The adopted analytical methods referred to statistical indicators such as the Gini coefficient, Thiel index, and agglomeration degree. Therefore, more comprehensive evaluation models for Chinese medical resource systems were lacking.

Therefore, the main objectives of this study were 1) to fill the above-mentioned gaps by adopting geographic big data of medical institutions and combining them with various statistical data to form a dataset of medical institutions in China; 2) to construct a medical resource scoring (*MRSc*) model based on the grading and classification of medical institutions by the Delphi method. The model was implemented at three levels (national, regional, and provincial) and the results were visualized in geospatial terms; 3) to summarize and discuss the clustering patterns of medical resources in China and further explore the relationship between medical resource scores (*MRSc*), population, and gross domestic product (GDP) based on geographically weighted regression (GWR) analysis. This study focused on the distribution pattern of medical resources in China, with the aim of providing a scientific basis for promoting the planned and equitable development of healthcare in China.

## Method

**Fig. 1:**
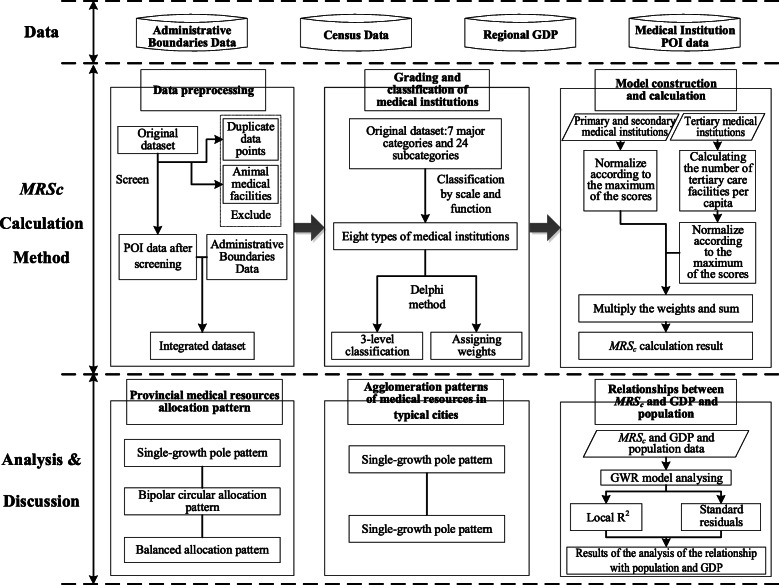
Main technical framework

In this study, an analytical technical framework for medical resources analysis technical framework, which containing data preparation, data preprocessing, grading and classification of medical institutions, and model construction and calculation, is proposed (Fig. 1).

### Data preparation

#### Base map and analysis units

The geographic base map applied in this study used vector data from the National Basic Geographic Database 2019, which is maintained by the National Geomatics Center of China. The vector layer does not contain any demographic data or other information but provides geographic entity codes (GEOIDs), provincial and municipal administrative codes, and provincial and municipal names for linking with other data (e.g., attribute datasets such as census data).

A total of 369 cities in China (excluding Taiwan) were used as the main analysis units in this study, including 278 prefecture cities, 30 province-administered counties, 30 autonomous regions, 15 sub-provincial cities, seven regions, four municipalities directly managed by the central government, three leagues, and two special administrative regions. These units are easily manageable in geographic information systems; this selection can adequately reflect spatial differences between regions.

#### Geographic big data for medical resources

“Medical resources” is the general term used to refer to factors of production that provide medical services. This term includes medical institutions, workforce, facilities, equipment, knowledge skills, and information [20]. The evaluation of medical resources in this study considers medical institutions as the primary focus. The large amount of medical geographic data in China provides a good basis for analyzing the spatial distribution and heterogeneity of medical resources in the country [21].

**Fig. 2:**
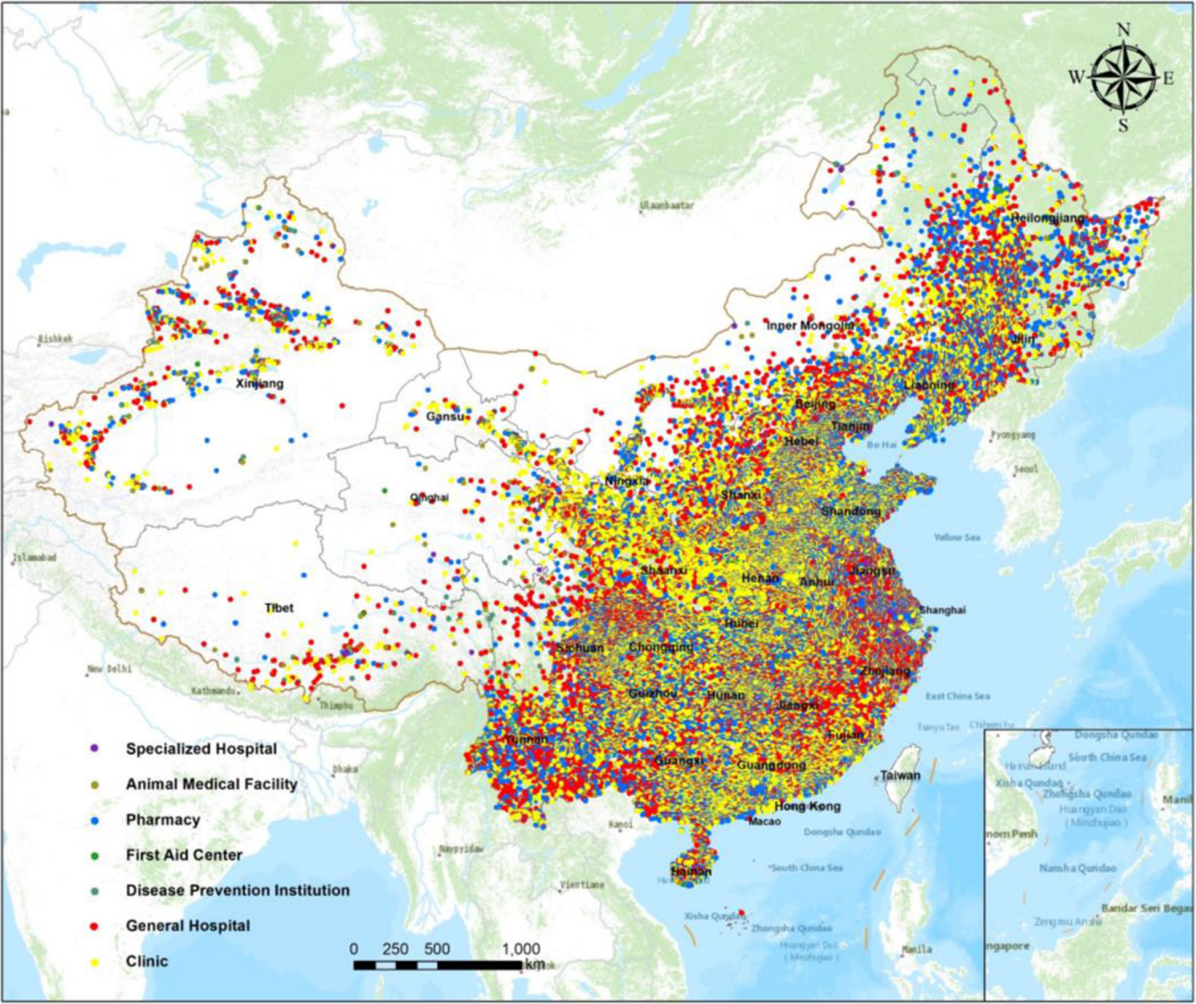
Study area and data point distributions. (Figure was generated using ArcGIS version 10.8 statistical software, URL: https://desktop.arcgis.com/en/system-requirements/latest/arcgis-desktop-system-requirements.htm)

The original dataset for this study was derived from multiple sources with heterogeneous formats and structures; therefore, we integrated each original dataset. The big data pertaining to the hospital institutions were sourced from Amap point data and represented as spatial points. They contain a total of 1,208,808 data points (Fig. 2), and the partial dataset is shown in Table 1.

**Table 1:** Excerpt of the dataset of medical institutions used in this study

Id	Medical Institution Name	Hospital Major Category	Hospital Subcategory	Longitude	Latitude	Province Code	Province Name	City Code	City Name	District Code	District Name
B0FFG96ECS	Beida Yanyuan Clinic	Clinic	Clinic	116.23388	40.199571	110,000	Beijing	10	Beijing	110,114	Changping District
B0FFMBBJJJ	Shengwang Hearing Aids	Pharmacy	Pharmacy	116.233614	40.224711	110,000	Beijing	10	Beijing	110,114	Changping District
B0FFHTMPRN	Huanrong Dental Hospital	Specialized Hospital	Dental Hospital	116.233288	39.84639	110,000	Beijing	10	Beijing	110,106	Fengtai District
B0FFHN13UX	No. 2 Qingta Rural Hospital	General hospital	Rural hospital	116.253561	39.895062	110,000	Beijing	10	Beijing	110,106	Fengtai District
B0FFF0E9HD	Aiguo Hospital	Specialized Hospital	Dental Hospital	116.241367	40.207911	110,000	Beijing	10	Beijing	110,114	Changping District
B000A85U1D	Xishan Community Rural Hospital	General hospital	Rural hospital	116.238296	39.955099	110,000	Beijing	10	Beijing	110,108	Haidian District

Moreover, 2019 city-based datasets were collected, mainly supplementing each city’s population and regional GDP data. These data were obtained from the 2019 Statistical Bulletin of National Economic and Social Development and China City Statistical Yearbook. The integrated dataset was formed and then added to the model for feature extraction and model analysis. We thus obtained the *MRS*_*c*_ table for each city. Fields such as province name, city name, city *MRS*_*c*_, and city medical resource ranking were retained in the table and linked to the base map.

### Medical resource scoring calculation method

#### Data preprocessing

The main reason for invalid points is data duplication. The dirty data in the original dataset are in the category of tertiary hospitals, in which there are multiple data points for the same hospital (different departments of one hospital), but they actually belong to one hospital.

The data points are filtered by distance, and the points within the 200-m buffer in the 3A hospitals are grouped into one data point. Chinese 3A hospitals were large-scale structures and not centrally distributed. The results of the screening are visually inspected in such a way as to ensure that there is no over-screening or under-screening.

After data screening, 31,539 data points referring to animal medical facilities and 1250 duplicate data points were excluded, leaving 1176,019 valid data points for the study.

#### Grading and classification of medical institutions

The original classification of the attributes of the medical institution dataset contains a total of seven major categories and 24 subcategories. Excluding the category of animal medical sites, the attributes were synthetically summarized into the following eight categories: 3A hospitals, general hospitals, specialized hospitals, disease prevention institutions, first aid centers, rural hospitals, clinics, and pharmacies. The Delphi method can be applied when a class of complex problems lacks a clear factual basis, and the subjective judgment of experts can help solve such problems [22]. In this study, the Delphi method was used to grade and assign weights to the above eight categories of healthcare organizations.

This study convened 111 healthcare experts from specific workplaces covering the above eight categories of medical institutions. An online format was used to conduct the survey, which ultimately yielded 105 valid responses and a response rate of 94.59%. Of the total samples, 74 used the 3-level classification, 22 used the 4-level classification, and nine used other classifications. When using the 3-level classification, 66 agreed with the weighting ratios of 0.6, 0.3, and 0.1, while 34 agreed with those of 0.5, 0.3, and 0.2.

The final expert opinion was as follows. China proposed a graded diagnosis and treatment reform program in 2015, and its medical resources were expressed in terms of high-quality medical resources and primary medical resources [23]. As shown in Table 2, according to their influence on and contribution to the healthcare system, the eight types of medical institutions mentioned above were classified into three levels, with weightings of 0.6, 0.3, and 0.1 at each level. Level I medical institutions consume the highest-quality medical resources in the region, provide high-level healthcare services, and are strongly equipped to serve the surrounding areas. They include 3A hospitals. Level II medical institutions comprise hospitals at the middle tier of the medical system. They are characterized by upward and downward referral functions that are less advanced and require lower skills than those of Level I medical institutions. Medical institutions with their own specific functions also appear in this category, as do general hospitals, specialized hospitals, disease prevention institutions, and first aid centers. Level III medical institutions are primary medical providers targeting urban and rural community residents with a small and relatively fixed scope of services. This level comprises rural hospitals, clinics, pharmacies. Notably, Hong Kong and Macau follow a hospital rating system separate from that of mainland China. As 3A hospitals are absent in this region, only secondary and tertiary categories were included in this grading system, resulting in a low score. Considering their different evaluation systems, Hong Kong and Macau will not be included in the scoring results and analysis.

**Table 2:** Medical institution grading table

Grade	Category	Weight	Number of data points	Percentage (%)
I	3A Hospital	0.6	1981	0.17
II	General hospital, specialized hospital, disease prevention institution, first aid center	0.3	172,983	14.71
III	Rural hospital, clinic, pharmacy	0.1	1,001,055	85.12

#### Medical resource scoring model

**Fig. 3:**
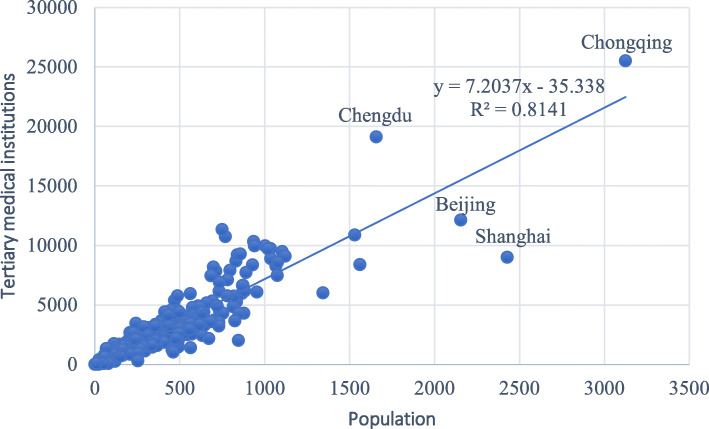
Correlation between the total number of tertiary medical institutions and the resident population served by each such institution

In this study, we defined a medical resource score (*MRS*_*c*_) for each city to reflect the abundance of medical resources at the municipal level in China, and the scores of the medical institutions at each level were normalized according to the maximum of the scores at each level. The total number of primary and secondary medical institutions visually reflects the richness of medical resources. As tertiary medical institutions are primary medical institutes it is not appropriate to directly use their quantities as an indicator to measure the richness of medical resources. However, Fig. 3 shows that the total number of tertiary medical institutions is positively correlated with the resident population (correlation coefficient = 0.81), implying a good fit. Thus, considering the prevalence of primary medical institutions in each city, the score of tertiary medical institutions in this scoring model serves as a per capita indicator. The model is mathematically described as follows:

1 $$ {S}_i=\frac{\sum {N}_{iu}}{\sum {N}_{iu\max }}\left(i=1,2\right) $$

2 $$ {S}_i=\frac{\sum {N}_{iu}/P}{\sum {N}_{iu\max }/{P}_{\mathrm{max}}}\left(i=3\right) $$

3 $$ {MRS}_C=\sum \limits_{i=1}^{i=3}{S}_i\times {W}_i $$

where *MRS*_*c*_ is the medical resource score of a city, *i* is the level of medical institution, *S*_*i*_ is the score of each level, *W* is the weight of each level, *u* is the category of the medical institutions in each level, ∑*N*_*iu*_ is the total number of medical institutions of all categories at a certain level, ∑*N*_*iu*max_ is the maximum total number of medical institutions of all categories at a certain level in each city, *P* is the permanent population of a city, and *P*_max_ is the permanent population of the city with the maximum number of medical institutions at a certain level.

### Scoring results analysis and evaluation method

#### GWR model used in this study

To explore the spatially varying relationship between *MRS*_*c*_ and population as well as *MRS*_*c*_ and GDP, this study used a GWR model. The GWR model considers parameters that vary geographically and spatially, in contrast to linear regression models. Application of a GWR to sampled data allows predictions by assigning classes of elements containing all explanatory variables for locations for which the dependent variable is unknown. Local parameters were used in the GWR model, which can be expressed with the following equations:

4 $$ {y}_i={\upbeta}_0\left({u}_i,{v}_i\right)+\sum \limits_{k=1}^p{\upbeta}_k\left({u}_i,{v}_i\right){x}_{k,i}+{\upvarepsilon}_i $$

where *y*_*i*_ is the dependent variable, *x*_*k*, *i*_ is the independent variable, ε_*i*_ is the random error term, (*u*_*i*_, *v*_*i*_) is the spatial location to which sample *i* belongs, and β_0_(*u*_*i*_, *v*_*i*_) is the model intercept.

The kernel type used a Gaussian weighted kernel function, which was expressed as follows:

5 $$ {w}_{ij}={e}^{\frac{-{d_{ij}}^2}{b^2}} $$

where *d*_*ij*_ is the Euclidean distance between the points, and *b* is the kernel bandwidth. In this study, the Akaike information criterion (AIC) was used to select the best bandwidth and the best model; models with lower AIC outperformed the other models [24].

#### Medical resource primacy ratio

The city primacy ratio can characterize the size of a city and its influence on its surroundings [25]. This work defines the medical resource primacy ratio as the ratio of *MRSc* of the primate city of each province to that of the secondary city. This ratio can represent the degree of concentration of medical resources in the primate city of each province to some extent, which was expressed as follows:

6 $$ R=\frac{M_1}{M_2} $$

where *R* is medical resource primacy ratio, *M*_1_ is the medical resources score of primate city; *M*_2_ is the medical resources score of secondary city.

#### Kernel density estimation

Kernel density estimation (KDE) is a method for solving the distribution density of a given sample set, which takes into account the decay effect of spatial distance. This method can effectively characterize the aggregated distribution of spatial entities, which is characterized by a closer distance to the core elements, larger kernel density value, and gradually decreasing kernel density value with increasing central radiation distance, and the spatial influence domain of geographic phenomena is characterized by the local space within the attenuation bandwidth. This is a nonparametric test to characterize the spatial influence domain of geographic phenomena through the local space within the attenuation bandwidth, which was expressed as follows:

7 $$ {f}_h(p)=\sum \limits_{i=1}^n\frac{1}{nh^2}k\left(\frac{p-{p}_i}{h}\right) $$

where *f*_*h*_(*p*) is the kernel density calculation function at position *p* in space; *h* is the bandwidth (also known as window width or smoothing parameter); *n* is the number of entities whose distance from position *p* is less than or equal to *h*; *k*(.) function represents the kernel probability density function, usually chosen with a variance of the standard Gaussian kernel function with the variance σ^2^. This work uses KDE to analyze the distribution and aggregation of high-quality medical resources (medical resources at levels I and II) in cities.

## Results

### Medical resource scoring

The 369 administrative units in China were scored according to the evaluation method explained in Section 3.2, and the results for the major cities are shown in Table 3 (the complete set of results appears in Additional file 1). The table showing the scoring results contains indicators such as city level, score, and ranking. The complete list of Chinese cities graded in 2019 was published by the First Financial Magazine. This list contained 337 cities divided into five grades, including four first-, 15 new first-, and 30 s-, 70 third-, 90 fourth-, and 128 fifth-tier cities. The city ranking framework included the following five indicators: business resource concentration, city pivotality, people activity, lifestyle diversity, and future malleability.

**Table 3:** Grading and results for the main cities in China

City Level	Province Name	City Name	Score	National Ranking	Provincial Ranking
First-tier city	Beijing	Beijing	92.89	1	1
First-tier city	Shanghai	Shanghai	67.54	2	1
First-tier city	Guangdong	Guangzhou	65.20	3	1
New first-tier city	Chongqing	Chongqing	65.05	4	1
New first-tier city	Sichuan	Chengdu	63.03	5	1
New first-tier city	Liaoning	Shenyang	50.19	6	1
New first-tier city	Zhejiang	Hangzhou	48.55	7	1
New first-tier city	Hubei	Wuhan	48.05	8	1
New first-tier city	Henan	Zhengzhou	44.18	9	1
New first-tier city	Tianjin	Tianjin	43.38	10	1
Second-tier city	Heilongjiang	Harbin	40.88	11	1
New first-tier city	Shaanxi	Xi’an	40.30	12	1
Second-tier city	Jilin	Changchun	38.96	13	1
New first-tier city	Shandong	Qingdao	38.10	14	1
First-tier city	Guangdong	Shenzhen	37.30	15	2
Second-tier city	Hebei	Shijiazhuang	37.18	16	1
Second-tier city	Liaoning	Dalian	36.86	17	2
New first-tier city	Hunan	Changsha	35.88	18	1
Second-tier city	Yunnan	Kunming	33.77	19	1
New first-tier city	Jiangsu	Nanjing	32.06	20	1

**Fig. 4:**
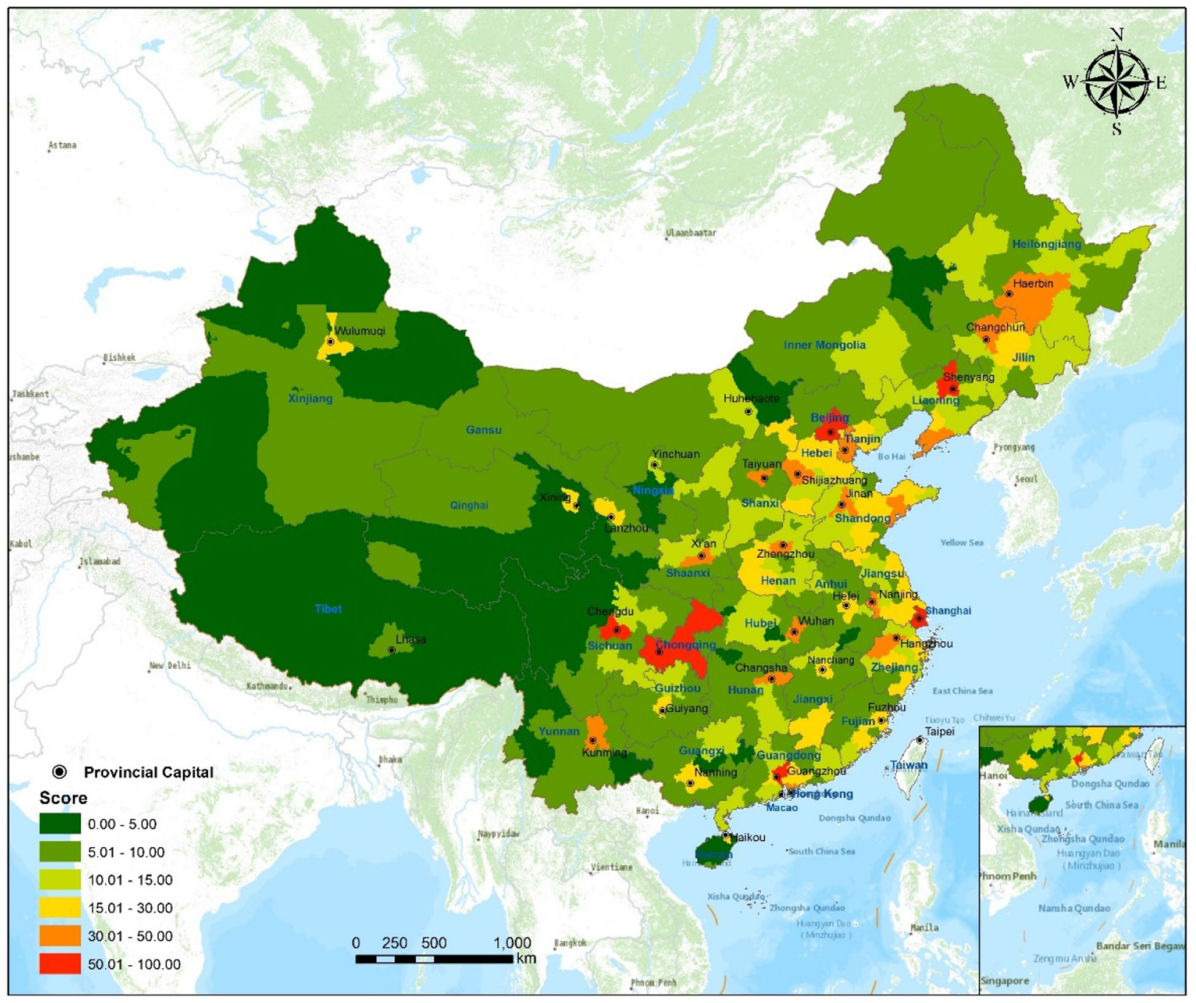
Spatial distribution of medical resource scores at the municipal level in China. (Figure was generated using ArcGIS version 10.8 statistical software, URL: https://desktop.arcgis.com/en/system-requirements/latest/arcgis-desktop-system-requirements.htm)

The results show that Beijing, Shanghai, and Guangzhou are ranked among the top three. In comparison, the top cities in Table 3 are primarily first- or new first-tier cities, roughly matching the city grading list. As shown in Fig. 4, the highly rated cities are generally distributed in the east, and most of the western cities are rated below 10.

### Medical resource primacy ratio

In general, a city primacy ratio of less than 2.0 indicates a normal structure and proper concentration; else, a tendency for structural imbalance and over-concentration exists. In this regard, 15 of China’s 27 provinces are found to be in a state of over-concentration, and the average value of the primacy ratio is 2.30, indicating that the spatial distribution of medical resources in China is unbalanced in each province and is generally concentrated in the primate city of each province. Among them, the first city in Sichuan Province in Chengdu, with a value of 5.11, has the highest concentration of medical resources, while Hohhot in Inner Mongolia shows the lowest value of 1.16 (Table 4).

**Table 4:** Rankings of the primacy ratio of medical resources in China’s major cities in each province

Province Name	Primacy Ratio of Medical Resources	Primate City	Secondary City
Sichuan	5.11	Chengdu	Mianyang
Hubei	3.96	Wuhan	Yichang
Yunnan	3.78	Kunming	Qujing
Xinjiang	3.58	Urumqi	Changji Hui Autonomous Prefecture
Heilongjiang	3.22	Harbin	Daqing
Shaanxi	3.07	Xi’an	Baoji
Tibet	2.72	Lhasa	Ngari Prefecture
Qinghai	2.61	Xining	Haixi Mongolian and Tibetan Autonomous Prefecture
Jilin	2.59	Changchun	Jilin
Hunan	2.45	Changsha	Hengyang
Hainan	2.34	Haikou	Sanya
Ningxia	2.19	Yinchuan	Shizuishan
Anhui	2.19	Hefei	Fuyang
Henan	2.11	Zhengzhou	Nanyang
Zhejiang	2.05	Hangzhou	Wenzhou
Guizhou	1.98	Guiyang	Zunyi
Gansu	1.96	Lanzhou	Qingyang
Shanxi	1.82	Taiyuan	Datong
Guangxi	1.78	Nanning	Guilin
Guangdong	1.75	Guangzhou	Shenzhen
Jiangsu	1.39	Nanjing	Xuzhou
Liaoning	1.36	Shenyang	Dalian
Hebei	1.36	Shijiazhuang	Baoding
Fujian	1.26	Fuzhou	Quanzhou
Jiangxi	1.24	Nanchang	Ganzhou
Shandong	1.19	Qingdao	Jinan
Inner Mongolia	1.16	Hohhot	Baotou

### Spatial allocation and equilibrium analysis of medical resources at different levels

#### Four economic sectors

According to the National Bureau of Statistics [26], China’s entire economic sector is divided into four parts: northeast, east, central, and west. The Northeastern Sector (NES) contains Liaoning, Jilin, and Heilongjiang. The Eastern Sector (ES) comprises Beijing, Tianjin, Hebei, Shanghai, Jiangsu, Zhejiang, Fujian, Shandong, Guangdong, Hainan, Hong Kong, and Macao. The Central Sector (CS) contains Shanxi, Anhui, Jiangxi, Henan, Hubei, and Hunan, while the Western Sector (WS) includes Inner Mongolia, Guangxi, Chongqing, Sichuan, Guizhou, Yunnan, Tibet, Shaanxi, Gansu, Qinghai, Ningxia, and Xinjiang. The scoring results are shown in Table 5.

**Table 5:** Scoring results of medical resource at the four economic sectors level

Economic Sector	Number of Units	Mean Score	Coefficient of Variation
NES	36	13.69	0.76
ES	89	15.08	0.98
CS	87	10.54	0.74
WS	141	7.87	1.11

**Fig. 5:**
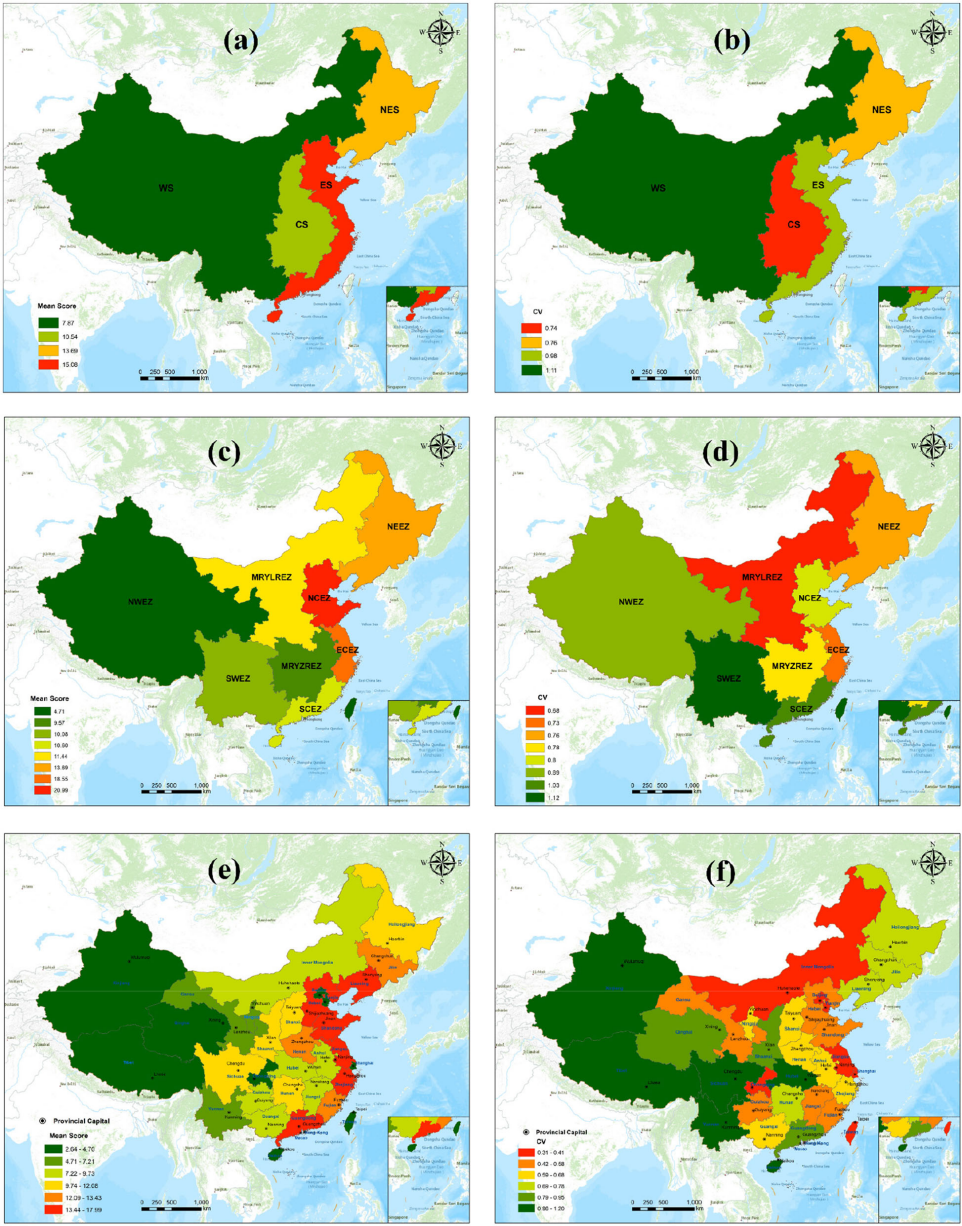
Spatial distribution of Chinese medical resources in three levels: **a** mean score of medical resource at the four economic sectors level, **b** CV of medical resource at the four economic sectors level, **c** mean score of medical resource at the eight economic zones level, **d** CV of medical resource at the eight economic zones level, **e** mean score of medical resource at the provincial level, and **f** CV of medical resource at the provincial level. (Figure was generated using ArcGIS version 10.8 statistical software, URL: https://desktop.arcgis.com/en/system-requirements/latest/arcgis-desktop-system-requirements.htm)

As shown in Fig. 5a and b, the mean score of medical care in the ES was significantly higher than that in the WS, whereas equalization was best in the CS and the worst in the WS.

#### Eight economic zones

During the 11th Five-Year Plan, the Development Research Center of the State Council proposed that the mainland economic zone be divided into eight economic zones. The first zone, the Northeast Economic Zone (NEEZ) comprises Liaoning, Jilin, and Heilongjiang. The Northern Coastal Economic Zone (NCEZ) contains Beijing, Tianjin, Hebei, and Shandong. The Eastern Coastal Economic Zone (ECEZ) includes Shanghai, Jiangsu, and Zhejiang, while the Southern Coastal Economic Zone (SCEZ) comprises Fujian, Guangdong, and Hainan. The Middle Reaches of the Yellow River Economic Zone (MRYLREZ) contains Shaanxi, Shanxi, Henan, and Inner Mongolia, while the Middle Reaches of the Yangtze River Economic Zone (MRYZREZ) comprises Hubei, Hunan, Jiangxi, and Anhui. The seventh zone, the Southwest Economic Zone (SWEZ) contains Yunnan, Guizhou, Sichuan, Chongqing, and Guangxi, while the last zone, the Northwest Economic Zone (NWEZ) covers Gansu, Qinghai, Ningxia, Tibet, and Xinjiang. The scoring results are shown in Table 6. As shown in Fig. 5c, d, the NCEZ and ECEZ exhibit the best medical conditions. The SWEZ shows the worst balance, while the MRYLREZ shows, the best balance, followed by the NEEZ and ECEZ.

**Table 6:** Scoring results of medical resource at the eight economic zones level

Economic Zone	Number of Units	Mean Score	Coefficient of Variation
NEEZ	36	13.69	0.76
NCEZ	29	20.99	0.80
ECEZ	25	18.55	0.73
SCEZ	49	10.60	1.03
MRYLREZ	51	11.44	0.68
MRYZREZ	58	9.57	0.78
SWEZ	61	10.08	1.12
NWEZ	58	4.71	0.89

#### Provinces

The provincial scoring results are shown in Table 7.

**Table 7:** Scoring results of medical resource at the provincial level

Province	Number of Units	Mean Score	Coefficient of Variation
Inner Mongolia	12	8.40	0.31
Jiangsu	13	15.93	0.41
Hebei	11	17.99	0.48
Shandong	16	17.16	0.50
Jiangxi	11	9.73	0.52
Gansu	14	6.83	0.53
Guizhou	9	9.01	0.57
Fujian	9	13.43	0.58
Anhui	16	8.67	0.60
Shanxi	11	11.62	0.63
Guangxi	14	9.15	0.64
Ningxia	5	6.94	0.67
Henan	18	13.01	0.67
Zhejiang	11	17.20	0.68
Hunan	14	10.67	0.72
Heilongjiang	13	11.92	0.76
Jilin	9	13.21	0.76
Liaoning	14	15.64	0.78
Guangdong	21	15.64	0.85
Shaanxi	10	12.08	0.86
Qinghai	8	4.70	0.95
Yunnan	16	7.21	1.02
Hainan	19	3.70	1.04
Tibet	7	2.64	1.05
Hubei	17	9.40	1.10
Sichuan	21	10.72	1.14
Xinjiang	24	3.62	1.20

As shown in Fig. 5e, the level of medical resources is generally higher in the eastern coastal regions than in the western inland regions. As for the medical level ranking by province, Guangdong, Jiangsu, Zhejiang, Shandong, and Hebei secure the top positions, while Xinjiang, Tibet, and Hainan show a low status.

As per Fig. 5f, the provinces with the worst equalization are located in the northwest and southwest; the central Hubei Province and the southernmost Hainan Province show poor equalization. The provinces with better equalization are situated in the northern area and coastal areas north of the Yangtze River.

Combining the results of the three levels shows a contradiction between the distribution of medical resources in the east-west and north-south areas. The distribution is concentrated along the eastern coast, in the ES (at the sector level), in the NCEZ and ECEZ (at the economic zone level), and in Zhejiang, Jiangsu, and Shandong (at the provincial level). Resource shortages and imbalanced focus areas are located in the west, the WS (at the economic sector level), the NWEZ (at the economic zone level), and Xinjiang, Tibet, and Qinghai (at the provincial level).

## Discussion

### Provincial medical resource allocation pattern

The medical resource allocation pattern refers to the spatial structure over time due to development at the province level after the accumulation of long-term regional experience. In this study, the patterns were grouped into three categories.

### Single-growth pole pattern

**Fig. 6:**
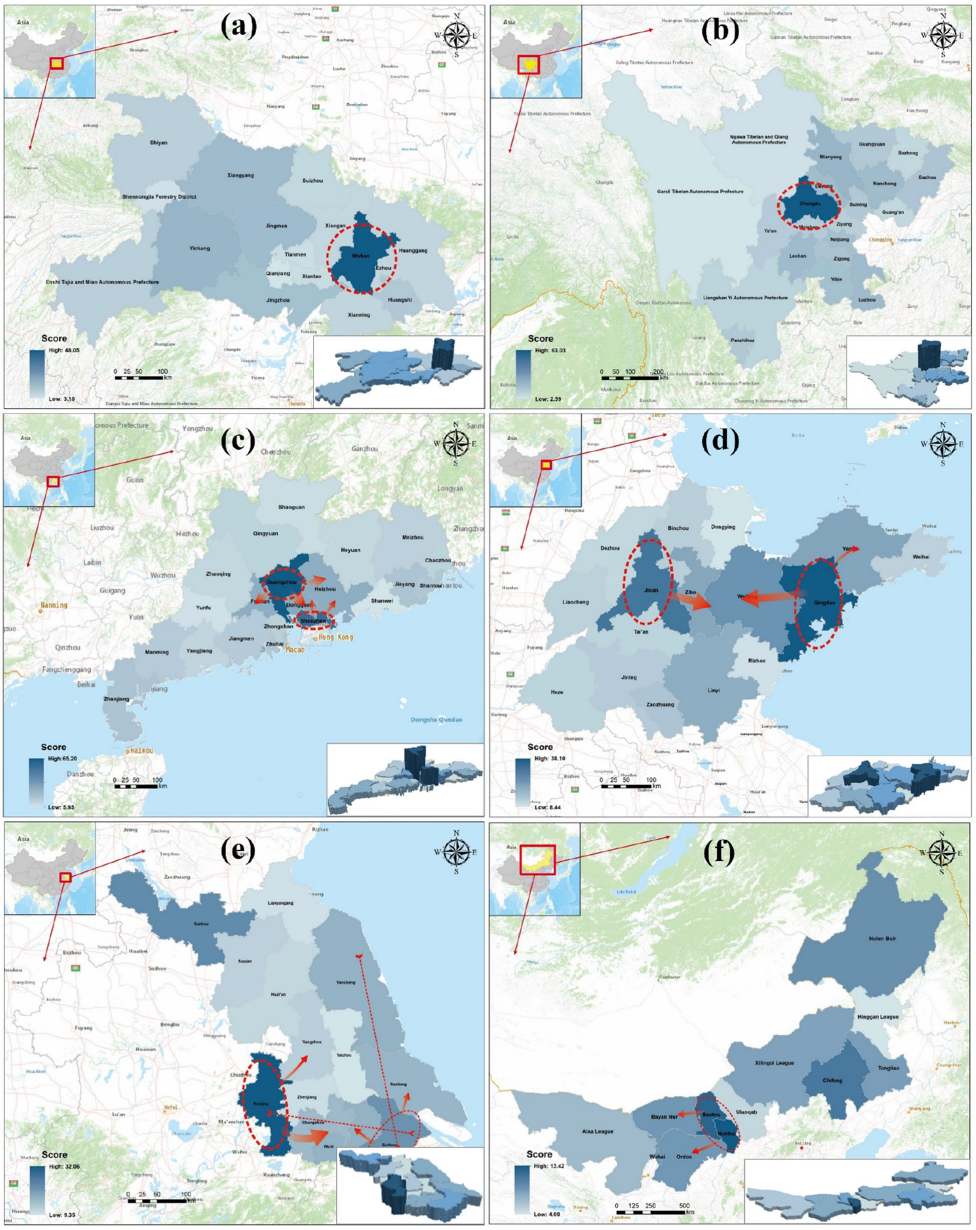
Spatial distribution of medical resources in China Provinces: **a** Hubei, **b** Sichuan, **c** Guangdong, **d** Shandong, **e** Jiangsu, and **f** the Inner Mongolia Autonomous Region. (Figure was generated using ArcGIS version 10.8 statistical software, URL: https://desktop.arcgis.com/en/system-requirements/latest/arcgis-desktop-system-requirements.htm)

The single-growth pole pattern refers to the pattern of a province that contains only one city with a relatively high level of medical resources. This pattern shows obvious characteristics of medical resource concentration, with the primate city exhibiting a much higher *MRS*_*c*_ than the other cities. Each province with this pattern suffers from a large gap at the medical level and a high degree of polarization. Two cities and provinces representative of this pattern are Wuhan in Hubei Province (Fig. 6a) and Chengdu in Sichuan Province (Fig. 6b). This pattern was found to have the broadest relative distribution in China, wherein the unipolar cities are the capital cities of their provinces. In addition to Sichuan and Hubei, 20 of the 27 provinces in China exhibit this pattern.

### Bipolar circular allocation pattern

The provinces depicting this configuration pattern are characterized by two centers of concentration and circular diffusion, as illustrated by Shandong and Guangdong. In Guangdong, the provincial capital city of Guangzhou and the sub-provincial city of Shenzhen form the innermost circle, which then expands to Dongguan, Huizhou, and Foshan, which form the central circle. The peripheral areas with lower concentrations of medical resources form the outermost circle (Fig. 6c).

Similarly, in Shandong, the provincial capital city, Jinan, and the sub-provincial city, Qingdao, form the innermost circle, which extends to Zibo, Weifang, Yantai, and Linyi (in the middle circle). Once again, the peripheral areas with low concentrations of medical resources form the outermost circle (Fig. 6d).

In this pattern, cities geographically located between the outermost and innermost circles, such as Dongguan and Weifang, enjoy unique location advantages, and their medical resource scores are usually higher in their respective provinces.

#### Balanced allocation pattern

In the balanced configuration mode, none of the cities are particularly outstanding relative to other municipalities, and the individual score does not deviate much from the average. This pattern includes Jiangsu and the Inner Mongolia Autonomous Region. The medical resources in these provinces are more divided, with Jiangxi and Inner Mongolia showing a lower overall level of medical care, while that in Jiangsu Province is almost the best in the country.

Jiangsu Province is located on the eastern coast of China. It is a developed region. The average *MRS*_*c*_ is 15.93, compared to the highest score of 32.06 for Nanjing and the lowest score of 9.35 for Taizhou. Eleven of 13 cities show a score of above 10, among which the Yangtze River basin cities and coastal cities show higher scores (Fig. 6e).

The Inner Mongolia Autonomous Region, located in the northernmost interior of China, has a mean score of 8.40; it is generally poor in medical resources, exhibiting a flat profile on the three-dimensional map seen in the inset (Fig. 6f). The highest score is 13.42 for Hohhot, and the lowest, 4.09, for Xing’an League, both of which are not too different from the average score.

### Agglomeration patterns of medical resources in typical cities

On the municipal scale, we selected Beijing, Shanghai, Chongqing, and Tianjin, four municipalities directly under the management of the central government of China, as typical cities to discuss the mode of medical resources collection. Each grade score of the three levels of medical institutions was normalized according to the highest value in each municipality directly under the central government, resulting in a medical resource score with the district as the evaluation unit. In addition, Beijing, Shanghai, Chongqing, and Tianjin are mega-cities, and given their internal high-quality medical resources, nuclear density analysis was carried out to explore the density distributions of their internal primary and secondary medical institutions. The final results are summarized as follows.

#### Single-center pattern

**Fig. 7:**
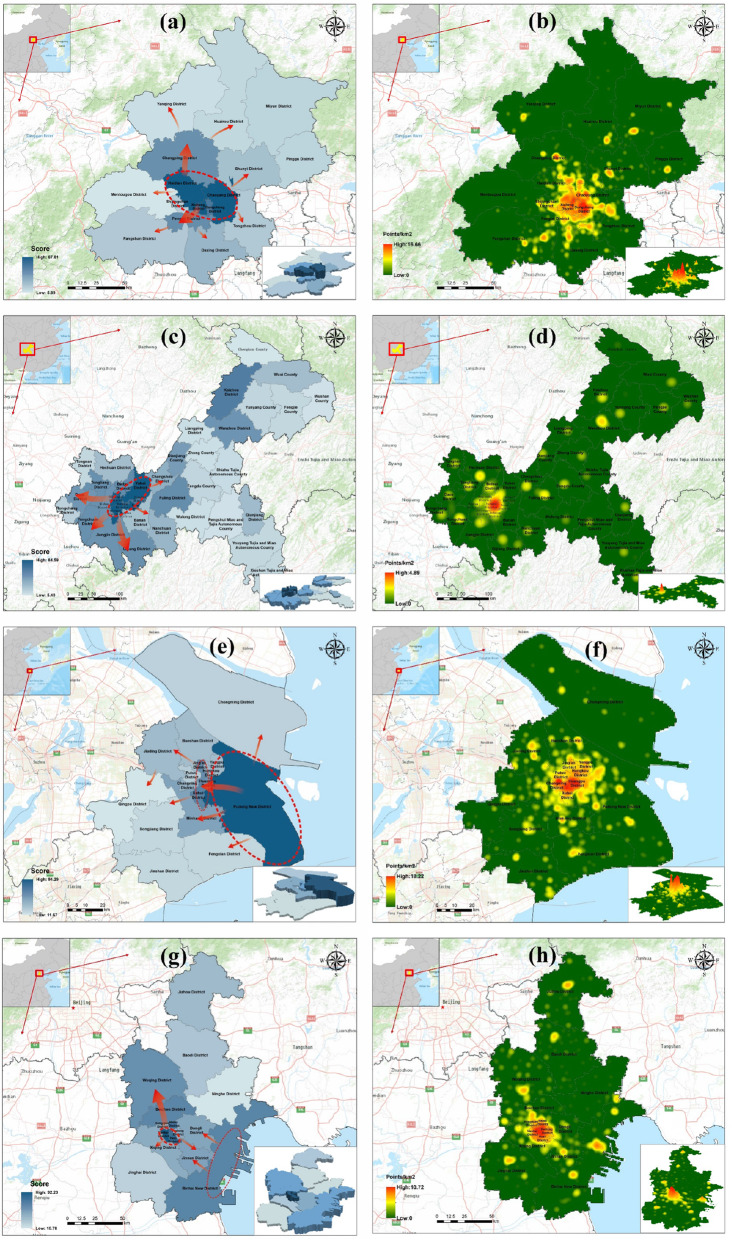
Spatial distribution of medical resources **a** by score in Beijing, **b** kernel-density distribution of high-quality medical resources in Beijing, **c** by score in Chongqing, **d** kernel-density distribution of high-quality medical resources in Chongqing, **e** by score in Shanghai, **f** kernel-density distribution of high-quality medical resources in Shanghai, **g** by score in Tianjin, and **h** kernel-density distribution of high-quality medical resources in Tianjin. (Figure was generated using ArcGIS version 10.8 statistical software, URL: https://desktop.arcgis.com/en/system-requirements/latest/arcgis-desktop-system-requirements.htm)

Beijing and Chongqing show a strong monocentric pattern for their respective overall medical resources, with a sharp decline from inside out. The highest concentration in Beijing is located in the three districts of Haidian, Chaoyang, and Xicheng, diffusing outward to the Changping, Dongcheng, and Fengtai districts, while the concentrations in the peripheral districts are relatively scarce (Fig. 7a). As shown in Fig. 7b, the highest densities of medical resources at levels I and II are concentrated in the city center, with the Chaoyang District showing the highest density, and the Changping District, a more pronounced concentration among the peripheral districts. The remainder are less dense.

The concentration of medical resources in Chongqing is located in the Yubei, Yuzhong, Shapingba, Jiulongpo, and Nan’an districts in the west of the city, and its outward diffusion mainly extends to the Bishan, Tongliang, and Jiangjin districts in the southwest (Fig. 7c). Thus, the quality of medical resources decreases outward, with the Yuzhong District as the center and the almost no high-density areas beyond it (Fig. 7d).

#### Balanced development pattern of old and new centers

In contrast to the previous pattern, the medical resources in Shanghai and Tianjin are located in both new and old city centers, each having developed into a medical service center for the region.

The distribution of medical resources in Shanghai is primarily concentrated in the Pudong New District and the old urban centers of the Jing’an, Xuhui and Huangpu districts. It then radiates outward to the Minhang, Baoshan, and Changning districts. Among them, high-quality medical resources and large- and medium-sized hospitals are still concentrated in the old city. These resources extend from the old city center to the southwest, to Pudong New District, forming a contiguous concentration area located northwest of the new area. No particular concentration areas are observed at the periphery of the two centers (Fig. 7e and f).

The distribution of medical resources in Tianjin is mainly concentrated in the Binhai New District and the old urban centers of the Nankai, Hexi, and Hebei districts (Fig. 7g). The old urban area delivers more medical resources to the north, while the Jinnan and Dongli districts, which are located between the old and new urban areas, are influenced by both these areas, exhibiting more medical resources and better future development prospects. Similarly, high-quality medical resources and large- and medium-sized hospitals are still concentrated in the old cities. Unlike Shanghai, the medical resources in the new center of Tianjin are not concentrated in a single direction, and several small concentrations scattered along the coast can be observed (Fig. 7h).

### Relationships between *MRS*_*c*_ and GDP and population

We analyzed the correlations between *MRS*_*c*_ and the GDP as well as *MRS*_*c*_ and the population using GWRs. In GWR, the normalized residuals are distributed over each geographical location. The optimal case, then, should present a perfect random distribution. We use Moran’s I to generate the parametric standardized residuals, and the test result was random. It shows the difference between this pattern and the random pattern does not seem to be significant, indicating that it passed the test.

**Fig. 8:**
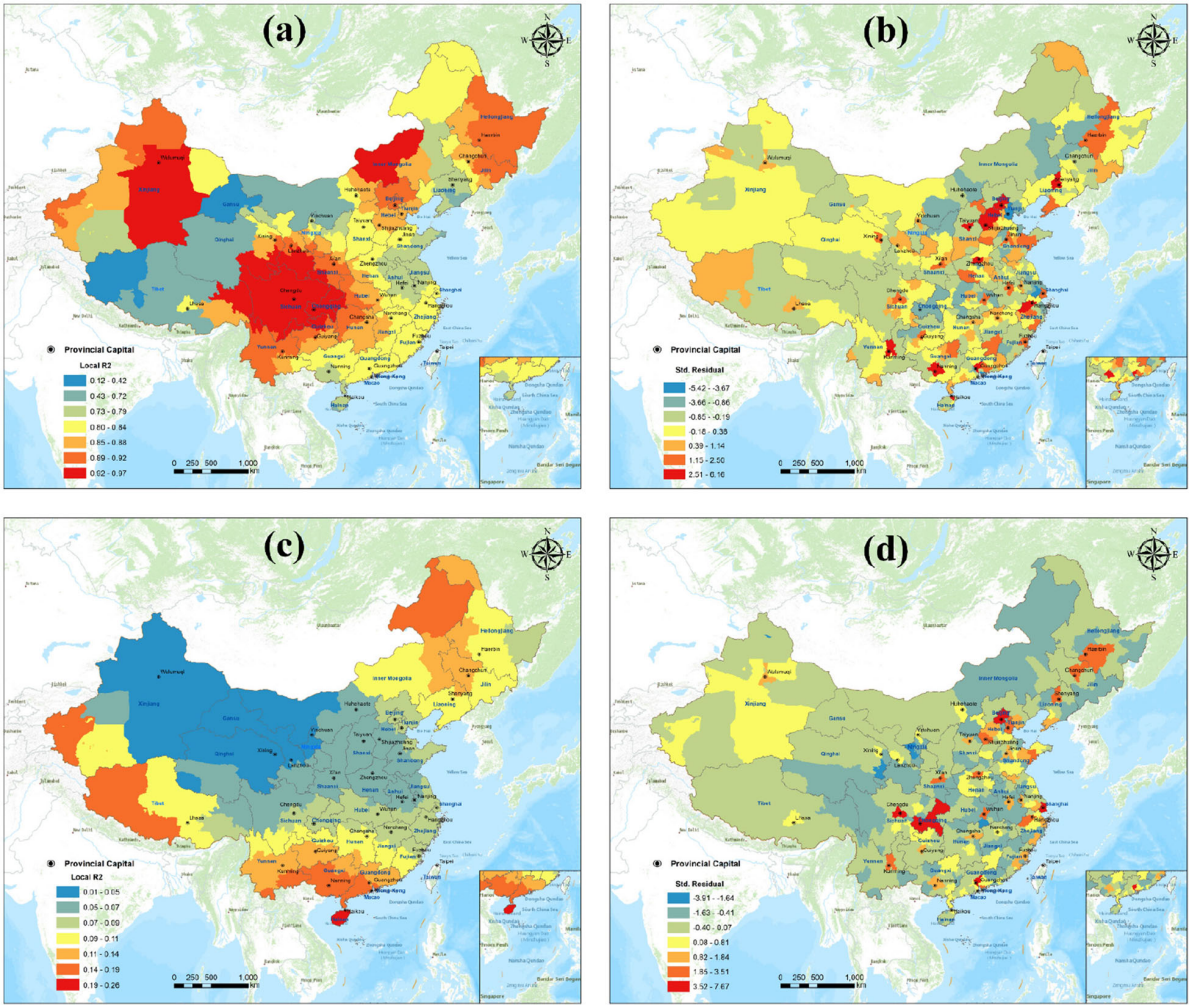
Relationship between medical resource scores and regional GDP and population: **a** Local R2 of medical resource scores and regional GDP, **b** standard residuals of medical resource scores and regional GDP, **c** local R2 of medical resource scores and population, and **d** standard residuals of medical resource scores and population. (Figure was generated using ArcGIS version 10.8 statistical software, URL: https://desktop.arcgis.com/en/system-requirements/latest/arcgis-desktop-system-requirements.htm)

As per Fig. 8a, the mean Local *R*^2^ is 0.83, and the *MRS*_*c*_ shows a good correlation with the regional GDP. The best correlation is noted in the northwest, northeast, and central regions. The sum of the standard residuals is 14.83. As shown in Fig. 8b, as per the GWR model, the actual *MRS*_*c*_ areis higher than the regression-predicted values for most regions when GDP is used to predict *MRS*_*c*_. This phenomenon is particularly evident in large cities with more developed economies, which have higher local fiscal revenues for healthcare resource investments and are also more appealing to capital, talent, and other factors, aspects that play an important role in improving local healthcare resource levels. However, the scores of the areas around big cities are lower than those obtained from the regression predictions, proving that their medical conditions do not match their actual economic development. We surmise that this result can be attributed to their distribution around big cities and mega-cities, caused by the strong medical resource aggregation capacity and polarization effect of such cities.

According to Fig. 8c, the mean local *R*^2^ is 0.09, and the *MRS*_*c*_ shows weak correlations with population, except for the relatively high Local *R*^2^ in the northeast. The overall trend correlation decreases from south to north. As shown in Fig. 8d, the sum of the standard residuals is − 5.75 in the GWR model. Population is used to predict the *MRS*_*c*_, and the actual *MRS*_*c*_ is lower than the regression prediction in most regions. The positive values appear for the provincial capitals and municipalities directly under the control of the central government. This indicates that most regions, especially the northeast and central regions, except for the provincial capitals and municipalities directly under the central government, still fall short of the medical resources needed to meet the needs of their corresponding populations.

In summary, medical resources in large cities match their GDP and population and are thus sufficient compared to those in the other regions. More work is required to ensure equitable distribution of these high-quality medical resources to the areas surrounding the big cities and solve the medical resource shortage in the western region of the country.

## Conclusion

Through the collection of geographic big data, statistical data and data filtering, a dataset of medical institutions applicable to China has been formed. Based on the dataset, this study established a medical resource evaluation model for China and defined the medical resource score (*MRS*_*c*_) as an indicator to measure the abundance of medical resources in different areas of the country. Using scoring and geospatial visualization of medical resources, the spatial distribution and balance of these resources were explored in three dimensions: the medical resources allocation patterns in Chinese provinces, the medical resource agglomeration patterns in typical Chinese cities, and the relationships between the medical scores, population, and GDP.

This study found that the eastern coastal region shows the highest medical resource abundance in China and that a decreasing trend from east to west is evident. The overall medical conditions in western China are poor and inequitable, and this shortage needs to be improved. In each province, the medical allocation pattern is dominated by a single-growth pole pattern, supplemented by the bipolar circular allocation and balanced allocation patterns. Medical resources are unevenly distributed in each province, with the top cities typically showing over-concentrations. Medical resources are also found to be highly correlated with regional GDP. Thus, policymakers should focus on using local finance to fill the medical resource gaps in each region and improve medical conditions in the peripheral areas of large cities to relieve the pressure on large cities in the near future. Subsequent studies should explore the differences in public demand for different types of medical resources at various levels. The distribution range and populations of various medical institutions at different levels should also be assessed to propose more fine-tuned strategies for optimizing medical resources allocation.

## Supplementary Information

**Additional file 1.** Complete China Medical Resources Scoring Result.

## Data Availability

The datasets used and analyzed during the current study are available from the corresponding author on reasonable request.

## Abbreviations

GDP: Gross domestic product
GWR: Geographically weighted regression
MRYLREZ: Middle Reaches of the Yellow River Economic Zone
MRYZREZ: Middle Reaches of the Yangtze River Economic Zone
SCEZ: Southern Coastal Economic Zone
NCEZ: Northern Coastal Economic Zone
ECEZ: Eastern Coastal Economic Zone
NEEZ: Northeast Economic Zone
NES: Northeast Sector
WS: Western Sector
CS: Central Sector
ES: Eastern Sector
