# Supplementary material for: Spatial analysis and evaluation of medical resource allocation in China based on geographic big data
Source: BMC Health Serv Res. 2021 Oct 12;21:1084. doi: 10.1186/s12913-021-07119-3 (PMC8508408; doi:10.1186/s12913-021-07119-3)
Supplement: Supplementary file 1 — Additional file 1. Complete China Medical Resources Scoring Result. [file 12913_2021_7119_MOESM1_ESM.docx]

## Appendix 1.

**Complete China Medical Resources Scoring Result.**

| Level | Province Name | Name | Score | National ranking | Provincial ranking |
| --- | --- | --- | --- | --- | --- |
| First-tier | Beijing | Beijing | 92.89 | 1 | 1 |
| First-tier | Shanghai | Shanghai | 67.54 | 2 | 1 |
| First-tier | Guangdong | Guangzhou | 65.20 | 3 | 1 |
| New First-tier | Chongqing | Chongqing | 65.05 | 4 | 1 |
| New First-tier | Sichuan | Chengdu | 63.03 | 5 | 1 |
| New First-tier | Liaoning | Shenyang | 50.19 | 6 | 1 |
| New First-tier | Zhejiang | Hangzhou | 48.55 | 7 | 1 |
| New First-tier | Hubei | Wuhan | 48.05 | 8 | 1 |
| New First-tier | Henan | Zhengzhou | 44.18 | 9 | 1 |
| New First-tier | Tianjin | Tianjin | 43.38 | 10 | 1 |
| Second-tier | Heilongjiang | Harbin | 40.88 | 11 | 1 |
| New First-tier | Shaanxi | Xi'an | 40.30 | 12 | 1 |
| Second-tier | Jilin | Changchun | 38.96 | 13 | 1 |
| New First-tier | Shandong | Qingdao | 38.10 | 14 | 1 |
| First-tier | Guangdong | Shenzhen | 37.30 | 15 | 2 |
| Second-tier | Hebei | Shijiazhuang | 37.18 | 16 | 1 |
| Second-tier | Liaoning | Dalian | 36.86 | 17 | 2 |
| New First-tier | Hunan | Changsha | 35.88 | 18 | 1 |
| Second-tier | Yunnan | Kunming | 33.77 | 19 | 1 |
| New First-tier | Jiangsu | Nanjing | 32.06 | 20 | 1 |
| Second-tier | Shandong | Jinan | 31.88 | 21 | 2 |
| Second-tier | Shanxi | Taiyuan | 30.99 | 22 | 1 |
| Second-tier | Fujian | Fuzhou | 28.09 | 23 | 1 |
| Second-tier | Hebei | Baoding | 27.42 | 24 | 2 |
| New First-tier | Anhui | Hefei | 26.65 | 25 | 1 |
| Second-tier | Guangxi | Nanning | 26.28 | 26 | 1 |
| Third-tier | Shandong | Weifang | 25.56 | 27 | 3 |
| Second-tier | Zhejiang | Wenzhou | 23.67 | 28 | 2 |
| Third-tier | Hebei | Handan | 23.62 | 29 | 3 |
| Second-tier | Jiangsu | Xuzhou | 23.08 | 30 | 2 |
| Second-tier | Fujian | Quanzhou | 22.31 | 31 | 2 |
| Third-tier | Xinjiang | Urumqi | 21.94 | 32 | 1 |
| Second-tier | Guizhou | Guiyang | 21.82 | 33 | 1 |
| New First-tier | Jiangsu | Suzhou | 21.54 | 34 | 3 |
| Second-tier | Shandong | Yantai | 21.40 | 35 | 4 |
| Second-tier | Zhejiang | Ningbo | 21.39 | 36 | 3 |
| Third-tier | Hebei | Tangshan | 21.22 | 37 | 4 |
| Third-tier | Shandong | Zibo | 21.05 | 38 | 5 |
| Second-tier | Jiangxi | Nanchang | 20.98 | 39 | 1 |
| Third-tier | Henan | Nanyang | 20.92 | 40 | 2 |
| New First-tier | Guangdong | Dongguan | 20.25 | 41 | 3 |
| New First-tier | Guangdong | Foshan | 19.85 | 42 | 4 |
| Third-tier | Shandong | Linyi | 19.78 | 43 | 6 |
| Second-tier | Guangdong | Huizhou | 19.76 | 44 | 5 |
| Second-tier | Fujian | Xiamen | 19.08 | 45 | 3 |
| Second-tier | Zhejiang | Jinhua | 18.45 | 46 | 4 |
| Second-tier | Hainan | Haikou | 18.44 | 47 | 1 |
| Third-tier | Henan | Luoyang | 18.02 | 48 | 3 |
| Second-tier | Gansu | Lanzhou | 17.78 | 49 | 1 |
| Fourth-tier | Shanxi | Datong | 17.06 | 50 | 2 |
| Third-tier | Jiangxi | Ganzhou | 16.94 | 51 | 2 |
| Third-tier | Hebei | Cangzhou | 16.75 | 52 | 5 |
| Fourth-tier | Liaoning | Yingkou | 16.66 | 53 | 3 |
| Second-tier | Jiangsu | Wuxi | 16.65 | 54 | 4 |
| Third-tier | Jiangsu | Yancheng | 16.63 | 55 | 5 |
| Second-tier | Hebei | Langfang | 16.30 | 56 | 6 |
| Second-tier | Jiangsu | Nantong | 16.24 | 57 | 6 |
| Second-tier | Jiangsu | Changzhou | 15.87 | 58 | 7 |
| Fourth-tier | Qinghai | Xining | 15.09 | 59 | 1 |
| Third-tier | Jilin | Jilin | 15.02 | 60 | 2 |
| Third-tier | Ningxia | Yinchuan | 14.96 | 61 | 1 |
| Second-tier | Zhejiang | Jiaxing | 14.91 | 62 | 5 |
| Third-tier | Guangxi | Guilin | 14.75 | 63 | 2 |
| Fourth-tier | Henan | Kaifeng | 14.72 | 64 | 4 |
| Third-tier | Hunan | Hengyang | 14.62 | 65 | 2 |
| Third-tier | Guangxi | Liuzhou | 14.61 | 66 | 3 |
| Fourth-tier | Shandong | Zaozhuang | 14.44 | 67 | 7 |
| Second-tier | Zhejiang | Shaoxing | 14.03 | 68 | 6 |
| Third-tier | Liaoning | Anshan | 13.81 | 69 | 4 |
| Fourth-tier | Hebei | Xingtai | 13.81 | 70 | 7 |
| Fourth-tier | Henan | Zhoukou | 13.79 | 71 | 5 |
| Third-tier | Guangdong | Zhanjiang | 13.73 | 72 | 6 |
| Third-tier | Guangdong | Jieyang | 13.67 | 73 | 7 |
| Third-tier | Shandong | Jining | 13.43 | 74 | 8 |
| Third-tier | Inner Mongolia | Hohhot | 13.42 | 75 | 1 |
| Fourth-tier | Jilin | Yanbian Korean Autonomous Prefecture | 13.24 | 76 | 3 |
| Fourth-tier | Shaanxi | Baoji | 13.12 | 77 | 2 |
| Fifth-tier | Shanxi | Changzhi | 12.86 | 78 | 3 |
| Fourth-tier | Shanxi | Yuncheng | 12.78 | 79 | 4 |
| Third-tier | Heilongjiang | Daqing | 12.70 | 80 | 2 |
| Third-tier | Shaanxi | Xianyang | 12.69 | 81 | 3 |
| Fourth-tier | Henan | Jiaozuo | 12.64 | 82 | 6 |
| Third-tier | Henan | Xinxiang | 12.60 | 83 | 7 |
| Fourth-tier | Shandong | Binzhou | 12.55 | 84 | 9 |
| Fourth-tier | Heilongjiang | Mudanjiang | 12.51 | 85 | 3 |
| Second-tier | Jiangsu | Yangzhou | 12.48 | 86 | 8 |
| Third-tier | Guangdong | Shantou | 12.47 | 87 | 8 |
| Fourth-tier | Henan | Anyang | 12.43 | 88 | 8 |
| Third-tier | Sichuan | Mianyang | 12.35 | 89 | 2 |
| Second-tier | Guangdong | Zhuhai | 12.23 | 90 | 9 |
| Third-tier | Jiangxi | Jiujiang | 12.19 | 91 | 3 |
| Third-tier | Anhui | Fuyang | 12.19 | 92 | 2 |
| Third-tier | Shandong | Weihai | 12.18 | 93 | 10 |
| Third-tier | Guangdong | Jiangmen | 12.18 | 94 | 10 |
| Fourth-tier | Guangdong | Maoming | 12.18 | 95 | 11 |
| Third-tier | Hubei | Yichang | 12.13 | 96 | 2 |
| Fifth-tier | Heilongjiang | Yichun | 12.11 | 97 | 4 |
| Fourth-tier | Heilongjiang | Jiamusi | 12.07 | 98 | 5 |
| Third-tier | Jiangsu | Suqian | 12.01 | 99 | 9 |
| Fourth-tier | Shandong | Liaocheng | 12.01 | 100 | 11 |
| Second-tier | Zhejiang | Taizhou | 11.99 | 101 | 7 |
| Third-tier | Shandong | Tai'an | 11.79 | 102 | 12 |
| Fifth-tier | Hebei | Hengshui | 11.77 | 103 | 8 |
| Fourth-tier | Shandong | Heze | 11.72 | 104 | 13 |
| Third-tier | Hunan | Chenzhou | 11.72 | 105 | 3 |
| Third-tier | Fujian | Zhangzhou | 11.72 | 106 | 4 |
| Fourth-tier | Shaanxi | Weinan | 11.64 | 107 | 4 |
| Third-tier | Inner Mongolia | Baotou | 11.59 | 108 | 2 |
| Fifth-tier | Liaoning | Fushun | 11.32 | 109 | 5 |
| Third-tier | Hebei | Qinhuangdao | 11.27 | 110 | 9 |
| Fourth-tier | Shanxi | Linfen | 11.26 | 111 | 5 |
| Fourth-tier | Liaoning | Jinzhou | 11.26 | 112 | 6 |
| Third-tier | Hubei | Xiangyang | 11.25 | 113 | 3 |
| Fourth-tier | Sichuan | Luzhou | 11.10 | 114 | 3 |
| Fourth-tier | Henan | Pingdingshan | 11.06 | 115 | 9 |
| Fourth-tier | Shandong | Dezhou | 11.05 | 116 | 14 |
| Third-tier | Guizhou | Zunyi | 11.04 | 117 | 2 |
| Fourth-tier | Sichuan | Nanchong | 11.01 | 118 | 4 |
| Fifth-tier | Jilin | Siping | 11.00 | 119 | 4 |
| Fifth-tier | Liaoning | Chaoyang | 10.98 | 120 | 7 |
| Fourth-tier | Henan | Zhumadian | 10.91 | 121 | 10 |
| Fourth-tier | Sichuan | Leshan | 10.89 | 122 | 5 |
| Fifth-tier | Hubei | Jingmen | 10.87 | 123 | 4 |
| Fourth-tier | Heilongjiang | Qiqihar | 10.87 | 124 | 6 |
| Third-tier | Jiangsu | Huai'an | 10.82 | 125 | 10 |
| Fourth-tier | Shaanxi | Yulin | 10.80 | 126 | 5 |
| Fourth-tier | Liaoning | Dandong | 10.65 | 127 | 8 |
| Third-tier | Guangdong | Meizhou | 10.62 | 128 | 12 |
| Fourth-tier | Sichuan | Deyang | 10.50 | 129 | 6 |
| Fourth-tier | Zhejiang | Quzhou | 10.40 | 130 | 8 |
| Fifth-tier | Jilin | Tonghua | 10.40 | 131 | 5 |
| Third-tier | Jiangsu | Zhenjiang | 10.38 | 132 | 11 |
| Fourth-tier | Inner Mongolia | Chifeng | 10.32 | 133 | 3 |
| Fifth-tier | Liaoning | Liaoyang | 10.30 | 134 | 9 |
| Fifth-tier | Henan | Puyang | 10.28 | 135 | 11 |
| Third-tier | Zhejiang | Huzhou | 10.27 | 136 | 9 |
| Second-tier | Guangdong | Zhongshan | 10.24 | 137 | 13 |
| Third-tier | Hubei | Jingzhou | 10.14 | 138 | 5 |
| Third-tier | Anhui | Ma on Shan | 10.12 | 139 | 3 |
| Third-tier | Hunan | Xiangtan | 10.10 | 140 | 4 |
| Third-tier | Guangdong | Chaozhou | 10.09 | 141 | 14 |
| Third-tier | Guangdong | Qingyuan | 10.02 | 142 | 15 |
| Fourth-tier | Sichuan | Yibin | 10.01 | 143 | 7 |
| Third-tier | Jiangsu | Lianyungang | 9.98 | 144 | 12 |
| Third-tier | Anhui | Bengbu | 9.89 | 145 | 4 |
| Third-tier | Hunan | Yueyang | 9.79 | 146 | 5 |
| Fourth-tier | Liaoning | Panjin | 9.77 | 147 | 10 |
| Fifth-tier | Liaoning | Huludao | 9.71 | 148 | 11 |
| Fourth-tier | Hebei | Zhangjiakou | 9.61 | 149 | 10 |
| Fourth-tier | Guangdong | Yangjiang | 9.58 | 150 | 16 |
| Fourth-tier | Guangxi | Yulin | 9.57 | 151 | 4 |
| Third-tier | Jiangxi | Shangrao | 9.54 | 152 | 4 |
| Fifth-tier | Sichuan | Neijiang | 9.39 | 153 | 8 |
| Third-tier | Jiangsu | Taizhou | 9.35 | 154 | 13 |
| Fourth-tier | Hunan | Changde | 9.34 | 155 | 6 |
| Third-tier | Zhejiang | Lishui | 9.26 | 156 | 10 |
| Third-tier | Fujian | Putian | 9.24 | 157 | 5 |
| Fifth-tier | Liaoning | Benxi | 9.24 | 158 | 12 |
| Fifth-tier | Liaoning | Fuxin | 9.23 | 159 | 13 |
| Third-tier | Guangdong | Zhaoqing | 9.21 | 160 | 17 |
| Fourth-tier | Shandong | Rizhao | 9.14 | 161 | 15 |
| Fifth-tier | Heilongjiang | Jixi | 9.10 | 162 | 7 |
| Fifth-tier | Gansu | Qingyang | 9.07 | 163 | 2 |
| Fifth-tier | Henan | Luohe | 9.02 | 164 | 12 |
| Fourth-tier | Jiangxi | Yichun | 9.02 | 165 | 5 |
| Fourth-tier | Yunnan | Qujing | 8.94 | 166 | 2 |
| Fifth-tier | Shaanxi | Yan'an | 8.93 | 167 | 6 |
| Fifth-tier | Liaoning | Tieling | 8.93 | 168 | 14 |
| Fourth-tier | Hebei | Chengde | 8.92 | 169 | 11 |
| Fourth-tier | Henan | Xuchang | 8.91 | 170 | 13 |
| Third-tier | Hunan | Zhuzhou | 8.91 | 171 | 7 |
| Third-tier | Anhui | Wuhu | 8.89 | 172 | 5 |
| Third-tier | Henan | Shangqiu | 8.86 | 173 | 14 |
| Fifth-tier | Inner Mongolia | Hulun Buir | 8.81 | 174 | 4 |
| Fifth-tier | Sichuan | Zigong | 8.78 | 175 | 9 |
| Fifth-tier | Sichuan | Dazhou | 8.76 | 176 | 10 |
| Fifth-tier | Inner Mongolia | Wuhai | 8.69 | 177 | 5 |
| Fifth-tier | Heilongjiang | Hegang | 8.69 | 178 | 8 |
| Fourth-tier | Guizhou | Anshun | 8.66 | 179 | 3 |
| Fourth-tier | Hubei | Huangshi | 8.65 | 180 | 6 |
| Third-tier | Fujian | Ningde | 8.60 | 181 | 6 |
| Fourth-tier | Tibet | Lhasa | 8.51 | 182 | 1 |
| Fifth-tier | Sichuan | Guangyuan | 8.49 | 183 | 11 |
| Fifth-tier | Heilongjiang | Qitaihe | 8.48 | 184 | 9 |
| Fourth-tier | Guangdong | Heyuan | 8.45 | 185 | 18 |
| Fourth-tier | Yunnan | Honghe Hani and Yi Autonomous Prefecture | 8.45 | 186 | 3 |
| Fourth-tier | Shandong | Dongying | 8.44 | 187 | 16 |
| Fifth-tier | Jilin | Songyuan | 8.42 | 188 | 6 |
| Fourth-tier | Hunan | Shaoyang | 8.37 | 189 | 8 |
| Fifth-tier | Inner Mongolia | Bayannaoer | 8.35 | 190 | 6 |
| Fourth-tier | Inner Mongolia | Ordos | 8.34 | 191 | 7 |
| Fifth-tier | Shanxi | Lvliang | 8.28 | 192 | 6 |
| Third-tier | Fujian | Sanming | 8.26 | 193 | 7 |
| Fourth-tier | Anhui | Suzhou | 8.18 | 194 | 6 |
| Fourth-tier | Hunan | Yongzhou | 8.17 | 195 | 9 |
| Fourth-tier | Guangdong | Shaoguan | 8.15 | 196 | 19 |
| Fifth-tier | Inner Mongolia | Tongliao | 8.08 | 197 | 8 |
| Fifth-tier | Sichuan | Panzhihua | 8.08 | 198 | 12 |
| Third-tier | Anhui | Chuzhou | 8.07 | 199 | 7 |
| Fifth-tier | Yunnan | Yuxi | 8.04 | 200 | 4 |
| Fifth-tier | Gansu | Jiayuguan | 8.03 | 201 | 3 |
| Fifth-tier | Sichuan | Suining | 8.03 | 202 | 13 |
| Fourth-tier | Guangxi | Beihai | 7.92 | 203 | 5 |
| Fourth-tier | Hunan | Huaihua | 7.91 | 204 | 10 |
| Third-tier | Hainan | Sanya | 7.90 | 205 | 2 |
| Fifth-tier | Jilin | Baishan | 7.89 | 206 | 7 |
| Fourth-tier | Guizhou | Qiannan Buyi and Miao Autonomous Prefecture | 7.87 | 207 | 4 |
| Fourth-tier | Hunan | Loudi | 7.85 | 208 | 11 |
| Fifth-tier | Inner Mongolia | Xilingol League | 7.83 | 209 | 9 |
| Fifth-tier | Guangxi | Guigang | 7.81 | 210 | 6 |
| Fifth-tier | Shanxi | Xinzhou | 7.79 | 211 | 7 |
| Fifth-tier | Sichuan | Ya'an | 7.78 | 212 | 14 |
| Fourth-tier | Heilongjiang | Suihua | 7.76 | 213 | 10 |
| Fifth-tier | Gansu | Baiyin | 7.73 | 214 | 4 |
| Fourth-tier | Shanxi | Jinzhong | 7.71 | 215 | 8 |
| Fifth-tier | Jiangxi | Pingxiang | 7.70 | 216 | 6 |
| Fourth-tier | Sichuan | Meishan | 7.63 | 217 | 15 |
| Fifth-tier | Anhui | Huaibei | 7.58 | 218 | 8 |
| Third-tier | Henan | Xinyang | 7.38 | 219 | 15 |
| Fifth-tier | Guizhou | Qianxinan Buyi and Miao Autonomous Prefecture | 7.33 | 220 | 5 |
| Fifth-tier | Guangdong | Yunfu | 7.29 | 221 | 20 |
| Fourth-tier | Guizhou | Qiandongnan Miao and Dong Autonomous Prefecture | 7.13 | 222 | 6 |
| Fourth-tier | Hubei | Enshi Tujia and Miao Autonomous Prefecture | 7.12 | 223 | 7 |
| Fifth-tier | Shanxi | Shuozhou | 7.05 | 224 | 9 |
| Fifth-tier | Guangxi | Qinzhou | 7.03 | 225 | 7 |
| Fifth-tier | Jilin | Baicheng | 7.02 | 226 | 8 |
| Fifth-tier | Henan | Hebi | 6.99 | 227 | 16 |
| Fourth-tier | Hubei | Shiyan | 6.96 | 228 | 8 |
| Fifth-tier | Jilin | Liaoyuan | 6.95 | 229 | 9 |
| Fifth-tier | Gansu | Pingliang | 6.93 | 230 | 5 |
| Fifth-tier | Yunnan | Chuxiong Yi Autonomous Prefecture | 6.92 | 231 | 5 |
| Third-tier | Fujian | Longyan | 6.91 | 232 | 8 |
| Fifth-tier | Ningxia | Shizuishan | 6.83 | 233 | 2 |
| Fifth-tier | Jiangxi | Xinyu | 6.82 | 234 | 7 |
| Fourth-tier | Hunan | Yiyang | 6.81 | 235 | 12 |
| Fifth-tier | Heilongjiang | Daxinganling region | 6.77 | 236 | 11 |
| Fourth-tier | Anhui | Bozhou | 6.75 | 237 | 9 |
| Third-tier | Anhui | Anqing | 6.72 | 238 | 10 |
| Fifth-tier | Sichuan | Ziyang | 6.71 | 239 | 16 |
| Third-tier | Fujian | Nanping | 6.65 | 240 | 9 |
| Fifth-tier | Gansu | Zhangye | 6.63 | 241 | 6 |
| Fifth-tier | Gansu | Tianshui | 6.58 | 242 | 7 |
| Fifth-tier | Inner Mongolia | Alxa League | 6.57 | 243 | 10 |
| Fifth-tier | Heilongjiang | Shuangyashan | 6.55 | 244 | 12 |
| Fourth-tier | Guangxi | Baise | 6.52 | 245 | 8 |
| Fifth-tier | Heilongjiang | Heihe | 6.47 | 246 | 13 |
| Fifth-tier | Guangxi | Fangchenggang | 6.45 | 247 | 9 |
| Fourth-tier | Guangxi | Wuzhou | 6.37 | 248 | 10 |
| Fifth-tier | Guangxi | Hechi | 6.33 | 249 | 11 |
| Third-tier | Zhejiang | Zhoushan | 6.30 | 250 | 11 |
| Fourth-tier | Jiangxi | Jingdezhen | 6.29 | 251 | 8 |
| Fifth-tier | Shaanxi | Hanzhong | 6.28 | 252 | 7 |
| Fourth-tier | Guizhou | Bijie | 6.27 | 253 | 7 |
| Fourth-tier | Jiangxi | Fuzhou | 6.24 | 254 | 9 |
| Fifth-tier | Gansu | Jinchang | 6.18 | 255 | 8 |
| Fifth-tier | Shanxi | Yangquan | 6.18 | 256 | 10 |
| Fourth-tier | Jiangxi | Ji'an | 6.16 | 257 | 10 |
| Fourth-tier | Anhui | Lu'an | 6.13 | 258 | 11 |
| Fifth-tier | Xinjiang | Changji Hui Autonomous Prefecture | 6.13 | 259 | 2 |
| Fourth-tier | Anhui | Huangshan | 6.12 | 260 | 12 |
| Not on the list | Yunnan | Dali Bai Autonomous Prefecture | 6.12 | 261 | 6 |
| Fifth-tier | Hunan | Xiangxi Tujia and Miao Autonomous Prefecture | 6.08 | 262 | 13 |
| Fifth-tier | Xinjiang | Bayinguoleng Mongolian Autonomous Prefecture | 6.07 | 263 | 3 |
| Fourth-tier | Hubei | Xianning | 6.06 | 264 | 9 |
| Fourth-tier | Yunnan | Xishuangbanna Dai Autonomous Prefecture | 6.02 | 265 | 7 |
| Fifth-tier | Henan | Sanmenxia | 6.01 | 266 | 17 |
| Fifth-tier | Sichuan | Liangshan Yi Autonomous Prefecture | 5.95 | 267 | 17 |
| Fourth-tier | Guangdong | Shanwei | 5.95 | 268 | 21 |
| Fifth-tier | Shaanxi | Ankang | 5.94 | 269 | 8 |
| Fifth-tier | Xinjiang | Ili Kazakh Autonomous Prefecture | 5.90 | 270 | 4 |
| Fourth-tier | Anhui | Xuancheng | 5.87 | 271 | 13 |
| Fourth-tier | Hubei | Huang Gang | 5.86 | 272 | 10 |
| Fourth-tier | Guizhou | Liupanshui | 5.85 | 273 | 8 |
| Fifth-tier | Shanxi | Jincheng | 5.80 | 274 | 11 |
| Fifth-tier | Gansu | Jiuquan | 5.78 | 275 | 9 |
| Fifth-tier | Qinghai | Haixi Mongolian and Tibetan Autonomous Prefecture | 5.77 | 276 | 2 |
| Fifth-tier | Shaanxi | Shangluo | 5.67 | 277 | 9 |
| Fifth-tier | Hubei | Suizhou | 5.60 | 278 | 11 |
| Fourth-tier | Anhui | Huainan | 5.58 | 279 | 14 |
| Fifth-tier | Xinjiang | Kashgar Prefecture | 5.56 | 280 | 5 |
| Fourth-tier | Hubei | Xiaogan | 5.50 | 281 | 12 |
| Fifth-tier | Guangxi | Chongzuo | 5.46 | 282 | 12 |
| Fifth-tier | Shaanxi | Tongchuan | 5.43 | 283 | 10 |
| Fifth-tier | Yunnan | Zhaotong | 5.43 | 284 | 8 |
| Not on the list | Henan | Jiyuan | 5.41 | 285 | 18 |
| Fifth-tier | Sichuan | Bazhong | 5.38 | 286 | 18 |
| Fourth-tier | Yunnan | Dehong Autonomous Prefecture of Dai and Jingpo Nationality | 5.34 | 287 | 9 |
| Fifth-tier | Hubei | Ezhou | 5.32 | 288 | 13 |
| Fourth-tier | Anhui | Tongling | 5.28 | 289 | 15 |
| Fifth-tier | Gansu | Dingxi | 5.26 | 290 | 10 |
| Fifth-tier | Sichuan | Guang'an | 5.20 | 291 | 19 |
| Fifth-tier | Gansu | Wuwei | 5.20 | 292 | 11 |
| Fourth-tier | Jiangxi | Yingtan | 5.18 | 293 | 11 |
| Fourth-tier | Guizhou | Tongren | 5.10 | 294 | 9 |
| Fifth-tier | Yunnan | Pu'er | 5.08 | 295 | 10 |
| Not on the list | Hainan | Qionghai | 4.99 | 296 | 3 |
| Fifth-tier | Guangxi | Hezhou | 4.96 | 297 | 13 |
| Not on the list | Hubei | Xiantao | 4.90 | 298 | 14 |
| Not on the list | Xinjiang | Shihezi | 4.79 | 299 | 6 |
| Fifth-tier | Ningxia | Zhongwei | 4.79 | 300 | 3 |
| Fifth-tier | Anhui | Chizhou | 4.71 | 301 | 16 |
| Fifth-tier | Inner Mongolia | Wulanchabu | 4.68 | 302 | 11 |
| Fifth-tier | Yunnan | Wenshan Zhuang and Miao Autonomous Prefecture | 4.48 | 303 | 11 |
| Not on the list | Hubei | Tianmen | 4.48 | 304 | 15 |
| Fifth-tier | Qinghai | Haibei Tibetan Autonomous Prefecture | 4.47 | 305 | 3 |
| Fifth-tier | Ningxia | Wuzhong | 4.15 | 306 | 4 |
| Fourth-tier | Yunnan | Lijiang | 4.12 | 307 | 12 |
| Fifth-tier | Inner Mongolia | Xing'an League | 4.09 | 308 | 12 |
| Fifth-tier | Gansu | Longnan | 4.06 | 309 | 12 |
| Fifth-tier | Ningxia | Guyuan | 4.00 | 310 | 5 |
| Fifth-tier | Guangxi | Laibin | 3.99 | 311 | 14 |
| Fifth-tier | Gansu | Linxia Hui Autonomous Prefecture | 3.87 | 312 | 13 |
| Fifth-tier | Xinjiang | Bortala Mongolia Autonomous Prefecture | 3.84 | 313 | 7 |
| Fifth-tier | Xinjiang | Hami | 3.83 | 314 | 8 |
| Fifth-tier | Xinjiang | Aksu Prefecture | 3.80 | 315 | 9 |
| Fifth-tier | Hunan | Zhangjiajie | 3.80 | 316 | 14 |
| Fourth-tier | Yunnan | Baoshan | 3.78 | 317 | 13 |
| Not on the list | Hubei | Shennongjia Forestry District | 3.74 | 318 | 16 |
| Fifth-tier | Qinghai | Hainan Tibetan Autonomous Prefecture | 3.67 | 319 | 4 |
| Not on the list | Sichuan | Aba Tibetan and Qiang Autonomous Prefecture | 3.54 | 320 | 20 |
| Not on the list | Hainan | Lingshui Li Autonomous County | 3.51 | 321 | 4 |
| Fifth-tier | Xinjiang | Tacheng Prefecture | 3.49 | 322 | 10 |
| Not on the list | Hainan | Wuzhishan | 3.46 | 323 | 5 |
| Not on the list | Hainan | Chengmai County | 3.39 | 324 | 6 |
| Fifth-tier | Yunnan | Lincang | 3.31 | 325 | 14 |
| Fifth-tier | Qinghai | Huangnan Tibetan Autonomous Prefecture | 3.23 | 326 | 5 |
| Not on the list | Xinjiang | Beitun | 3.16 | 327 | 11 |
| Fifth-tier | Qinghai | Haidong | 3.15 | 328 | 6 |
| Fifth-tier | Xinjiang | Karamay | 3.15 | 329 | 12 |
| Fifth-tier | Tibet | Ngari prefecture | 3.13 | 330 | 2 |
| Not on the list | Hubei | Qianjiang | 3.10 | 331 | 17 |
| Fifth-tier | Xinjiang | Turpan | 2.96 | 332 | 13 |
| Not on the list | Hainan | Wanning | 2.93 | 333 | 7 |
| Fifth-tier | Tibet | Linzhi | 2.89 | 334 | 3 |
| Not on the list | Hainan | Wenchang | 2.88 | 335 | 8 |
| Fifth-tier | Yunnan | Diqing Tibetan Autonomous Prefecture | 2.76 | 336 | 15 |
| Fifth-tier | Yunnan | Nujiang Lisu Autonomous Prefecture | 2.74 | 337 | 16 |
| Not on the list | Hainan | Baoting Li and Miao Autonomous County | 2.72 | 338 | 9 |
| Fifth-tier | Sichuan | Ganzi Tibetan Autonomous Prefecture | 2.59 | 339 | 21 |
| Fifth-tier | Gansu | Gannan Tibetan Autonomous Prefecture | 2.48 | 340 | 14 |
| Not on the list | Hainan | Dongfang | 2.47 | 341 | 10 |
| Fifth-tier | Hainan | Danzhou | 2.31 | 342 | 11 |
| Not on the list | Xinjiang | Wujiaqu | 2.29 | 343 | 14 |
| Not on the list | Xinjiang | Tumushuk | 2.27 | 344 | 15 |
| Not on the list | Hainan | Ding'an County | 2.25 | 345 | 12 |
| Not on the list | Hainan | Qiongzhong Li and Miao Autonomous County | 2.14 | 346 | 13 |
| Not on the list | Hainan | Tunchang County | 2.11 | 347 | 14 |
| Fifth-tier | Hainan | Sansha | 2.06 | 348 | 15 |
| Fifth-tier | Xinjiang | Altay Prefecture | 1.83 | 349 | 16 |
| Not on the list | Hainan | Baisha Li Autonomous County | 1.81 | 350 | 16 |
| Not on the list | Hainan | Changjiang Li Autonomous County | 1.76 | 351 | 17 |
| Not on the list | Hainan | Ledong Li Autonomous County | 1.68 | 352 | 18 |
| Not on the list | Hainan | Lingao County | 1.49 | 353 | 19 |
| Fifth-tier | Xinjiang | Hotan Prefecture | 1.47 | 354 | 17 |
| Not on the list | Xinjiang | Alar | 1.41 | 355 | 18 |
| Fifth-tier | Tibet | Shigatse | 1.37 | 356 | 4 |
| Fifth-tier | Qinghai | Yushu Tibetan Autonomous Prefecture | 1.21 | 357 | 7 |
| Fifth-tier | Xinjiang | Kizilsu Kirgiz Autonomous Prefecture | 1.19 | 358 | 19 |
| Fifth-tier | Tibet | Shannan | 1.06 | 359 | 5 |
| Fifth-tier | Qinghai | Golog Tibetan Autonomous Prefecture | 1.04 | 360 | 8 |
| Fifth-tier | Tibet | Naqu | 0.80 | 361 | 6 |
| Fifth-tier | Tibet | Qamdo | 0.69 | 362 | 7 |
| Not on the list | Xinjiang | Huyanghe | 0.49 | 363 | 20 |
| Not on the list | Xinjiang | Shuanghe | 0.46 | 364 | 21 |
| Not on the list | Xinjiang | Kirkdala | 0.42 | 365 | 22 |
| Not on the list | Xinjiang | Tiemenguan | 0.27 | 366 | 23 |
| Not on the list | Xinjiang | Kunyu | 0.17 | 367 | 24 |
